# Retention of a resin-based sealant and a glass ionomer used as a fissure sealant in children with special needs

**DOI:** 10.4317/jced.51688

**Published:** 2014-12-01

**Authors:** Mariana C. Morales-Chávez, Zacy-Carola Nualart-Grollmus

**Affiliations:** 1 Pediatric Dentist. Magister in Special Care Dentistry. Aggregate Professor and Director of the Dental Research Center. Santa Maria University. Caracas, Venezuela; 2 Pediatric Dentist. Magister in Special Care Dentistry. Assistant Professor of Mayor University, Temuco, Chile

## Abstract

Objectives: The aim of this research is to evaluate the retention of sealants of resin and resin-modified ionomeric glass pits and fissures, on first permanent molars of special patients. Material and Methods: The sample was comprised by 32 children. The ages were between 7 and 18 years. The sealing procedure was made with the relative isolation of the molars to be sealed, through the use of cotton rolls. Two molars were sealed with Clinpro Sealant 3M Dental and the others with Vitremer. Checking of the sealants was made after 3 and 6 months of their placement, evaluating with 3 values: TR: Totally Restrained; PR: Partially Restrained; and CL: Completely Lost. Results: 67.18% of the resinous sealants, and 70.31% of the glass ionomer sealants were successful after three months. After six months, 57.81% of the resin-based sealants and 51.56% of the glass ionomer sealants were successful. When performing the Chi-square statistical analysis (P<0.05) no statistical significance was observed after 6 months. Conclusions: The retention of the resin sealant was similar to that of the glass ionomer cement at the end of six months and the retention of sealants on maxillary teeth was higher than on mandibular teeth.

** Key words:**Sealant, glass ionomer, retention, caries, special needs.

## Introduction

According to the World Health Organization, there are over a billion people in the world living with some kind of disability. Almost 200 million of them experience considerable difficulties to function. Many systemic diseases, disabling conditions, and medical treatments may reduce the caries resistance of the patient. Actually, these patients are more prone to suffering from caries due to motor coordination problems that hinder or difficult mouth hygiene, lack of care on the side of the people that look after them, ingestion of a big number a medications that favor xerostomy, and excessively bland diets (1).

The use of sealants on pits and fissures is still one of the most widely accepted methods by the new cavity-prevention programs, as 80% of them develop in the pits and fissures of the tooth, due to the favorable conditions for the accumulation of plaque, and because it is a place in which fluoride cannot act. Sealants must be placed between 1 – 1.5 years post eruption, as this is the period considered to be critical, because the enamel is not completely ripe (2-9).

Several clinical studies have shown that resin-based sealants that have been used since their introduction in the market in 1965 by Cueto and Buonocore, are nothing but a physical barrier that prevents the metabolic exchange between microorganisms of pits and fissures, and the mouth environment. Also, the application of resin-based sealants is a very humidity-sensitive technique, in which contamination could be equal to treatment failure. This very common contamination in the mouth cavity is very hard to control in special patients, because of the impossibility of collaboration on the side of the patient (10-12).

In 1974, glass ionomers were introduced by Mclean and Wilson. They have the peculiarity of releasing fluoride in the tooth and saliva, even a year after it has been placed (10,13). It was then proposed as a sealant for pits and fissures in not very cooperative children, in hard to isolate teeth, in semi-erupted first molars, or as a transitional sealant (9,11).

Unlike the short effect on the dental enamel of topically applied fluoride, glass ionomer sealants trigger a spreading mechanism by which mouth fluid anions are attracted by the opposite charges, performing an exchange with the fluoride, spreading it to the surface and liberating it. This mechanism allows for proper physical properties and fluoride release from days up to years, decreasing the occurrence of caries after the acid attack up to 35%, and is even capable of reducing demineralization to a few millimeters of the material (14,15).

The literature has reported a decrease in enamel solubility and artificial secondary caries with fluoride dental materials, preventing demineralization and promoting mineralization (14).

Another advantage of using glass ionomers as sealants is the decrease in work time, as the acid does not need to be etched to achieve the chemical bonding to the tooth. This time, in the case of patients with disabilities, is of vital importance, as in most cases work is very complicated, and sometimes physical restraints are necessary (6,13).

Although a lower retention of glass ionomer sealants has been reported, compared to resin-based sealants, the caries prevention effect is significantly higher with the ionomer, as it releases important concentrations of fluoride that penetrate up to 60 µm into the tooth enamel. However, with the development of resin modified photo-polymerizable ionomers, this disadvantage has been minimized (2,6,13,14).

The aim of this research is to evaluate the retention of sealants of resin and resin-modified ionomeric glass pits and fissures, on first permanent molars of special patients.

## Material and Methods

This study was made at a Dentistry Center for Disabled People in Valencia, Spain. The evaluations, placement, and reviewing of the sealants were performed between January – December, 2006. The materials used in this study were Clinpro Sealant 3M Dental, resin sealants and Vitremer, 3M Dental, a resin-modified photopolymerizable glass ionomer. These materials were used on the first four permanent molars in a collateral manner, in order to diminish the saliva contamination variable, due to the higher isolation difficulties in some areas.

Patients were selected randomly from the total population of children that attended the office during a previous period of three months, at the beginning of the study. The inclusion criteria included the psychological disability diagnosis of the patient, the presence of the four permanent molars - completely erupted and with no cavities, in a post-eruptive period not higher than two years -, the absence of bruxism, and an informed consent from the legal guardian. The children with hypoplastic permanent first molars or developmental anomalies were excluded from the study.

The sample was comprised by 32 children - 10 girls [28.1%] and 22 boys [71.9%]. The ages of the patients were between 7 and 18 years, and the average age was 10.25. In many cases, the age of the patients was higher than six years, as patients with disabilities’ can show eruption delays of up to two years.

The study was conducted according to a full mouth design using contralateral teeth. The sealing procedure was made with the relative isolation of the molars to be sealed, through the use of cotton rolls. The occlusal surfaces were cleaned with a prophylaxis brush, and fluoride-free paste. Two molars were sealed with the resin-based sealant, with the previous etching with phosphoric acid 35%, for 30 seconds and then remove by using air-water spray. After that, the operator the dry the etched enamel and apply the sealant. Using the syringe needle tip or a brush, slowly introducing sealant into the pits and fissures. Finally, the sealant was cured by exposing it to light from light curing unit. A 20-second exposure was needed for each surface.

Molars sealed with the ionomer were prepared with the conditioner, which was photopolymerized for 10 seconds while the dental assistant mixed the product Using a cement spatula, mixed the powder into the liquid. All of the powder should be incorporated into the liquid within 45 seconds. Then the ionomer was placed in a dry field and cured by exposing its entire surface area to 40 seconds of visible light curing unit. Finally, the finishing shine was applied. The glass proportion was altered by the operators to 1 powder measure for every 2 liquid drops, to get a more fluid texture [de Luca-Fraga *et al.* technique]. The applications of the fissure sealants was performed by one previously calibrated operator and a dental assistant. The re-examination in three and six month was also performed by one previously calibrated operator and a dental assistant. The intra-examiner variability was minimized by reexamination of 15% of patients. The kappa coefficient for intra-evaluator consistency was 0.89 and 0.91 respectively.

In order to perform this procedure, it was necessary to use a retractor, physical restraints, or premedication with Diazepam, and also behavioral management for many patients. In two patients only the retractor was used; on 4 it was necessary to use physical restraints. On other 10 patients, it was necessary to use both retractor and physical restraints. On six patients, premedication with Diazepam was required, either orally or rectal, five of which also needed physical restraints.

Checking of the sealants was made after 3 and 6 months of their placement, evaluating with 3 values: P: Present; PP: Partially Present; and L: Lost. Lost sealants were not placed again during the study. The presence or absence of caries was also evaluated in the case of the molars with the sealant completely lost.

Finding on retention of the two materials were tabulated according to the molar and length of follow-up period. The data were analyzed using the SPSS 17.0 statistics program for Windows at the 5% significance level. Chi square was applied to determinate statistical significance.

## Results

In the study, 32 handicapped patients were selected, 5 autistic patients [15.62%], 9 with Down syndrome [28.12%], 6 with cerebral palsy [18.75%], and 12 with slight to moderate mental retardation [37.5%]. The patients were distributed amongst two groups; 11 physically and psychologically challenged patients [34.4%], and 21 exclusively with psychological disability [65.6%]. It was determined that 90.6% followed a normal diet, and 9.4% a bland diet. 25% of the patients were treated with behavioral management, 6.3% also required the use of a retractor, 50% of the patients needed physical restraints, and 18.8% was prescribed with oral benzodiazepines.

Retention levels were evaluated considering 3 criteria: present, partially present, and lost ( Table 1). The clinical condition of the materials used was evaluated after three and six months. After three months, 65.62% of the resin-based sealants were present, as well as 70.31% of the glass ionomer sealants. On the other hand, 21.87% of resinous sealants, and 25% of glass ionomer sealants had been lost. In terms of the position of the tooth, 64% of the upper pieces showed a totally retained sealant, regardless of the material used; unlike lower pieces, in which the sealant was found totally retained on 48.43% of the cases.

**Table 1:**
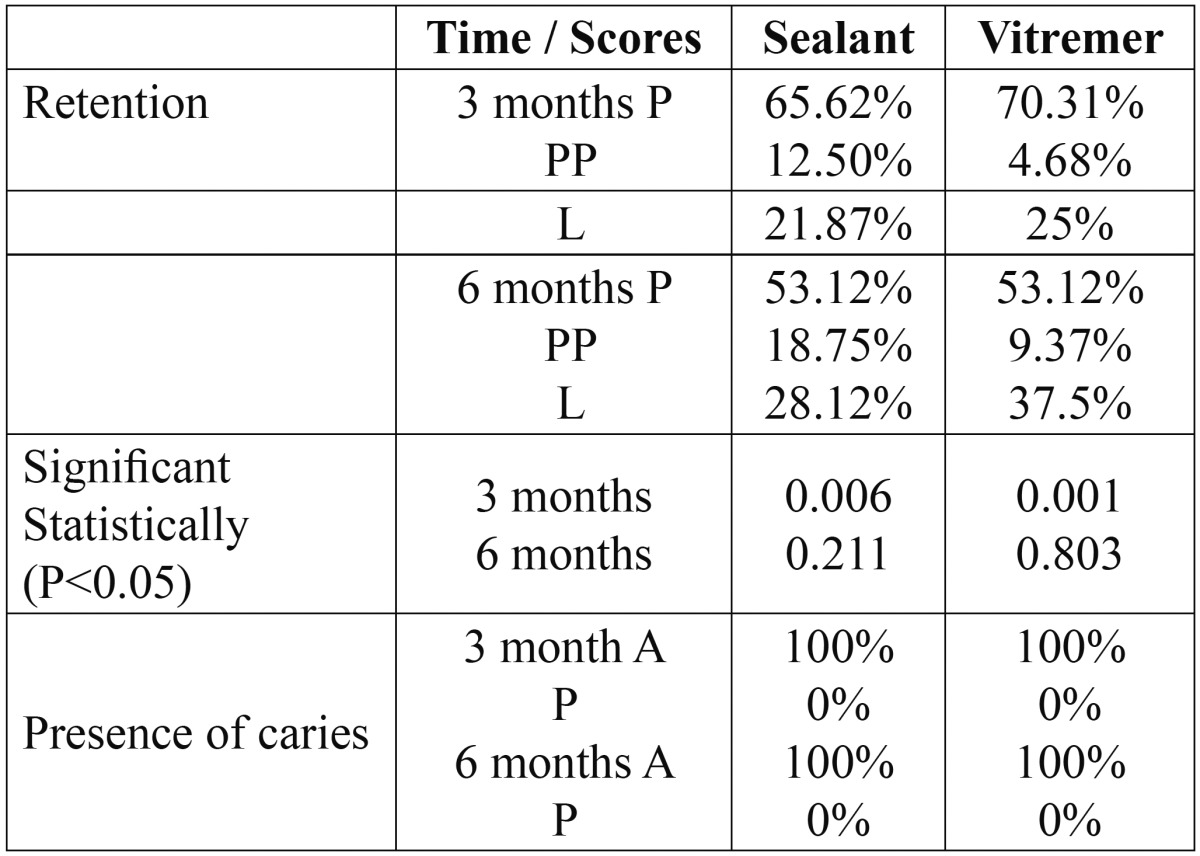
Evaluation of retention levels considering present, partially present, and lost criteria.

In terms of the presence of caries, none of the teeth, even if they had lost the sealant, showed caries during the clinical exam ( Table 1).

Besides the evaluation of retention based on the aforementioned criteria [present, partially present, and lost], the data was separated in two groups [success, failure], where only sealants found to be completely present were considered a success, and the other two criteria were considered failures. With this analysis it was determined that 67.18% of the resinous sealants and 70.31% of the glass ionomer sealants were successful after three months. After six months, 57.81% of the resin-based sealants and 51.56% of the glass ionomer sealants were successful ( Table 2).

**Table 2:**
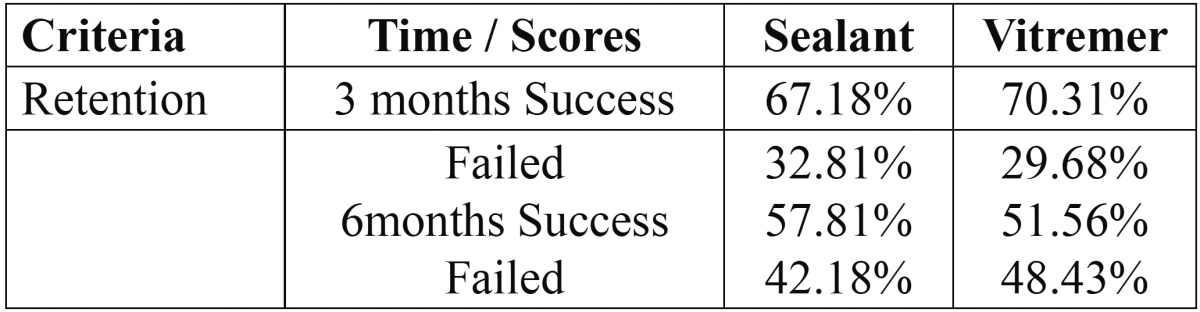
Analysis of the data separated in two groups: success and failure.

When performing the Chi-square statistical analysis [*P*<0.05] it was determined that there was a statistically significant relationship, where the glass ionomer was more effective. However, no statistical significance was observed after 6 months.

## Discussion

There are few studies comparing two materials to seal pits and fissures in patients with special needs.

Dental sealants have been proved to be highly effective in the prevention of pit and fissure caries. The caries preventive property of sealants is based on the placement of a seal that prevents nutrients from reaching the microflora in the fissure (4). Many researchers have confirmed that ionomeric glass has many advantages if used as a sealant for pits and fissures in recently erupted teeth, as it has similar effects in terms of caries prevention; however, it is easier to handle, and the etching with acid is not necessary (4).

This study compared the clinical success of a resin-based and glass ionomer sealants, used to seal pits and fissures on permanent first molars of 32 handicapped children, which were evaluated after 6 months.

In our study, at the end of the 6 months period, resin-based sealants showed a total retention of 53.12%, partial retention of 18.75%, and 28.12% had been completely lost. In terms of the ionomer glass sealants, this study determined that 53.12% were present, 9.37% were partially retained and 37.5% were absent. Poulsen *et al.* (12) performed a study in which they compared the retention of a resin-based sealant with a glass ionomer sealant, and found that, after 6 months, 90.09% of the resin-based sealants were completely retained, 6.75% were partially retained, and 3.15% had been completely lost. Glass ionomer sealants were found to be present in 13.06% of the cases, partially retained 38.10% of the times, and absent in 50% of the teeth. Similar results were obtained by Forss *et al.* (13), after comparing the retention of a resin-based sealant, and of a glass ionomer cement. The results were 10.3% of the glass ionomer sealants, and 45.5% of the resin-based sealants were totally present, showing a statistically significant retention difference. Also, Subramaniam *et al.* (4) determined a retention percentage of 38.3% after 6 months for resin sealants, and 13.1% for glass ionomer sealants. These results show a higher retention in the teeth in which a resin-based sealant was used; unlike our study, in which the results were similar for both materials after 6 months, although after 3 months statistical differences were found, with a higher retention for Vitremer sealants. Likewise, Guler *et al.* (10) found a higher retention after 6 months for glass ionomer sealants, with 82%, versus 73% for resin sealants. Pardi *et al.* (9) evaluated the clinical retention of two glass ionomer sealants [Vitremer and Ketac-Bond], and found a higher retention with Vitremer with the passing of time: an evaluation after 2 years found 14.2% present, compared to 3.5% of Ketac Bond.

Regarding caries prevention, in our study, clinical evaluation after six months determined the absence of caries in 100% of the teeth, even though many of them had lost the sealant. Nevertheless, it is important to remember that the time of follow-up of six months is a little time to evaluate the effect on the appearance of new caries. Guler *et al.* (10) found the presence of caries in 3% of the teeth treated, regardless of the sealant used. Kervanto *et al.* (16) compared the caries prevention effect on two types of sealants in a group of children of ages ranging from 12 to 16 years, and determined a statistically significant difference, in terms of caries prevention as they found that resin-based sealants were more effective than their glass ionomer counterpart. Also, Poulsen *et al.* (12) found a higher incidence of caries in teeth that were sealed with glass ionomer cement.

The possible reasons for failure of a resin sealant can be poor placement technique [inadequate etching, rinsing or drying, and insufficient curing time], the position of the tooth in the mouth, the skill of the operator, and the handicap of the patient. On the other hand, the main reason for the loss of the glass ionomer sealants could be inadequate adhesion of the cement to the enamel surface, the difficulty to isolate in handicapped patients, or excessive salivation (4).

## Conclusions

Dental caries prevention in handicapped patients is almost uncharted territory in the field of odontology, probably due to the same lack of early attention of these patients. Many times, when they finally make it to the dentist’s office, it is already time to perform more aggressive treatments. However, nowadays, and due to a better spreading of information, parents go earlier with their children to a dentist’s appointment, which allow us to act in a preemptive instead of a curative manner. Besides the basic measures of mouth hygiene, such as brushing, the use of dental floss, and topical fluoride, the dentist has to perform periodic examinations, and implement the use of preemptive non-invasive techniques, such as the topic application of fluoride, and sealants to pits and fissures.

The following conclusions were drawn from the study:

• The retention of the resin sealant was similar to that of the glass ionomer cement at the end of six months.

• The retention of sealants on maxillary teeth was higher than on mandibular teeth.
